# Association between incorrect postures and curve magnitude of adolescent idiopathic scoliosis in china

**DOI:** 10.1186/s13018-024-04767-z

**Published:** 2024-05-17

**Authors:** Xiaosheng Chen, Yongyu Ye, Zhixiang Zhu, Rui Zhang, Weijun Wang, Miaoling Wu, Xinhai Lu, Bin Yan, Qian Liang

**Affiliations:** 1 Department of spine surgery, Shenzhen Second People’s Hospital, the First Affiliated Hospital of Shenzhen University, Sungang west road, Futian district, Number 3002, Shenzhen, 518035 China; 2 Shenzhen Youth Spine Health Center, Shenzhen, China; 3 Department of Orthopedic Surgery, Guangdong Provincial People’s Hospital, Southern Medical University, Guangzhou, China

**Keywords:** Adolescent, Scoliosis, Screening, Incorrect postures, Curve magnitude

## Abstract

**Background:**

Despite advancements in school scoliosis screening (SSS), there are still no effective indicators to estimate the severity of spinal curvature. We aim to investigate the association between incorrect postures and curve magnitude of adolescent idiopathic scoliosis (AIS) among Chinese adolescents.

**Methods:**

In this SSS program, we examined the incorrect posture, Adam’s forward bending test (FBT) results, and angle of trunk rotation (ATR) in adolescents. Those with suspected scoliosis were referred for a standing anteroposterior whole-spine radiography as outpatients. The radiographic data of 426 students with lateral Cobb angles were collected from 2016 to 2022 and the associations were studied using logistic regression (LR) models and receiver operating characteristic (ROC) curves.

**Results:**

Univariate LR revealed that female gender [odds ratio (OR) = 2.92, 95% confidence interval (CI) 1.67–5.09, *P* < 0.001], age 16–19y (OR = 2.83, 95%CI 1.10–7.28, *P* = 0.031), right shoulder height (OR = 2.15, 95%CI 1.23–3.75, *P* = 0.007), right scapula tilt (OR = 2.03, 95%CI 1.18–3.50, *P* = 0.010), right rib hump (OR = 1.88, 95%CI 1.23–2.85, *P* = 0.003), right thoracic rotation ≥ 5° (OR = 2.14, 95%CI 1.43–3.20, *P* < 0.001), and left thoracolumbar kyphosis (OR = 3.79, 95%CI 1.06–13.56, *P* = 0.041) were all significantly associated with the severity of the curve magnitude. Multivariate LR showed that female gender [adjusted OR (AOR) = 3.23, 95%CI 1.81–5.73, *P* < 0.001], those aged 16–19y (AOR = 5.08, 95%CI 1.86–13.91, *P* = 0.002), and with a right rib hump (AOR = 1.72, 95%CI 1.11–2.64, *P* = 0.015) presented with a higher risk of severe curve magnitude than men, those aged 7–12y, and without a rib hump, respectively. ROC curves further proved that sex, age, shoulder-height difference, scapula tilt, flat back, rib hump, angle of thoracic rotation were the risk predictors for curve magnitude.

**Conclusion:**

Incorrect posture and ATR, especially the right rib hump, were significantly associated with the curve magnitude of AIS. Early screening for incorrect postures and ATR could be an effective and economical strategy to predict the severity of AIS through SSS in Chinese adolescents.

**Supplementary Information:**

The online version contains supplementary material available at 10.1186/s13018-024-04767-z.

## Introduction

Adolescent idiopathic scoliosis (AIS) is a complex three-dimensional spinal deformity, defined by a lateral Cobb angle of ≥ 10° that generally develops in early puberty without a discernable cause [1]. AIS affects 0.5–5.2% of adolescents in early puberty, with a higher prevalence in women [2–4]. Most patients with a mild curvature are asymptomatic during adolescence, and this condition is overlooked by teachers and parents. However, if not treated early, scoliosis can cause curve progression, cosmetic deformity, back pain, functional limitations, respiratory compromise, and psychological complications [1, 5, 6]. Moreover, this disease aggravates more rapidly and severely in adolescents and requires surgical intervention when accompanied with severe curvatures, exerting a significant economic burden on the family and society [7, 8]. Despite advancements in treatment, early intervention, including exercise and bracing, is the most effective treatment for relatively small curves [9]. Thus, school scoliosis screening (SSS) has been used to detect students at risk of scoliosis before curvature progression to allow prompt intervention [4].

In China, SSS generally comprises visual examination for physical signs, performance of Adam’s forward bending test (FBT), and measurement of the angle of trunk rotation (ATR) with a scoliometer [4]. Schoolchildren deemed at risk were referred to undergo a second screening and whole-spine radiography, followed by diagnosis and treatment based on the Cobb angle [9–11]. Currently, there are significant issues with SSS, such as the low positive predictive value (PPV), high time and money costs involved, problems with the optimal age for scoliosis screening, and the unnecessary radiography caused by over-referral [1, 9, 12]. Thus, it is necessary to screen for indicators to accurately identify patients with AIS. Scoliometers have been approved for their validity and reliability in measuring the ATR for scoliosis screening [13]. In addition, evidence has shown that multiple physical signs jointly evaluated by screening staff may increase the PPV of SSS in detecting scoliosis [14, 15]. According to our previous study, 65.3% of students in a Chinese cohort (*n* = 595,057) were found to have incorrect postures (including shoulder-height difference, scapula tilt, and pelvic tilt) [16]. An incorrect posture refers to an improper state in which the body cannot maintain equilibrium in an upright posture [17]. Nault et al. [15] and Stylianides et al. [18] both found that asymmetrical postures were more pronounced in female adolescents with scoliosis than that in healthy controls. Our previous studies further proved that incorrect postures were significantly associated with the occurrence of AIS in SSS [19, 20]. Standing incorrectly could also result in spinal pain in adolescents, which may be an early sign of scoliosis progression [15, 21]. Overall, the current studies supported that the postural changes in body attitude were linked to scoliosis. However, whether these main school screening items, including incorrect postures and ATR, are associated with and emerge as reliable predictors for the severity of AIS remains unclear.

The aim of this study was to test the hypothesis that early monitoring for incorrect postures associated with spinal curvature in adolescents and school adolescents should be considered as an effective intervention to identify the scoliosis severity. Therefore, we collected data from 426 individuals who were suspected of having scoliosis based on our SSS for AIS in China and had total spine X-rays as outpatients. We analyzed current school screening items, including sex, age, incorrect postures, and ATR stratified by major curve magnitude, and analyzed the potential association between these screening items and AIS severity. Our results will help to comprehensively identify indicators of the severity of spinal curvature to improve the effectiveness of SSS in China.

## Methods

### Subjects and school scoliosis screening (SSS) program

Data were collected from our Chinese School based Scoliosis Screening Program (CSSSP), which is part of the national public health project targeted at Chinese children and adolescents (age, 6–18y) admitted in primary, junior high, and senior high schools in Shenzhen. The SSS program is conducted and administered by the Shenzhen Youth Spine Health Center (SYSHC) at the Shenzhen Second People’s Hospital, in accordance with a nationally standardized protocol (GB/T16133-2014). This study was approved by the Ethics Committee of Shenzhen Second People’s Hospital and all participants signed informed consent to participate. Scoliosis screening was conducted in schools by a team of experienced rehabilitation therapists from the SYSHC using visual examination, Adam’s FBT, and a scoliometer to measure ATR. Students suspected of having AIS were referred for a radiograph to determine the degree of spinal curvature.

Individuals with a clinical diagnosis of neuromuscular scoliosis or congenital scoliosis were excluded from the study. Data from 426 students who underwent spinal radiographs and had their periodic outpatient follow-up file were collected from 2016 to 2022 at the outpatient clinic.

### Measurements

According to our previous large-scale population-based study investigating 595,057 students [16], in addition to other evidence [9], the combined use of multiple clinical signs of incorrect posture could improve the ability of the PPV to detect AIS. Thus, to explore the potential predictors and establish an accurate prediction model of severity of AIS, the measurement variables used in the study were drawn from the demographic information, multiple signs of incorrect postures, and ATR of students to determine the potential predictors of spinal curvature severity. The demographic characteristics included sex (male or female) and age (years). Incorrect posture and ATR were assessed via visual examination, Adam’s FBT, and scoliometer measurements. The standard visual examination was performed in an upright position. The screening staff inspected students for spine alignment, shoulder asymmetry (e.g., shoulder-height difference), scapular prominence (e.g., scapular tilt), hip and pelvic obliquity (e.g., pelvic tilt), thoracic curvature (e.g., flat back, thoracic kyphosis), thoracolumbar curvature (thoracolumbar kyphosis), and lumbar curvature (e.g., lumbar concave, lumbar kyphosis). Next, the Adam’s FBT was performed with the students’ feet together, knees straight, hips bending to 90°, arms hanging freely forward, and palms facing each other. A scoliometer was then used to measure the angles of thoracic rotation, thoracolumbar rotation, and lumbar rotation. Students who participated in the screening were separately examined by two independent therapists. To minimize subjective bias, a third examiner made the final judgment if the results were inconsistent. More detailed methods and procedures for SSS have been described in our previous studies [16, 19, 20].

When students were diagnosed with one or more significant physical symptoms of scoliosis or an ATR > 5°, they were reexamined by specially trained physicians, and further recommended to undergo whole-spine anteroposterior radiograph in the standing position for final diagnoses at the outpatient clinic. Scoliosis was diagnosed on measuring the lateral Cobb angles of the main curve by two independent, experienced observers.

### Statistical analysis

Descriptive analyses (Mann-Whitney U test or Kruskal-Wallis test) were used to analyze the demographic characteristics, prevalence of incorrect postures, and ATR among students stratified by major curve magnitude (Cobb angle < 10°, 10–19°, 20–39°, ≥ 40°). Of these, comparisons between two groups (sex) were assessed using the Mann-Whitney U test, and three or more independent groups (age, incorrect postures, and ATR) were compared with the Kruskal-Wallis test. The PPV was calculated by dividing the number of diagnosed cases by the number of referrals from screening. Univariate ordinal logistic regression (LR) models were used to preliminarily determine the correlation between screening items (sex, age, incorrect postures, ATR) and curve magnitude. Multivariate ordinal LR was performed to identify the independent effects of each sign of incorrect postures on the major curve. From the LR models, the odds ratios (ORs), adjusted odds ratios (AORs), and 95% confidence intervals (CIs) were obtained. In addition, receiver operating characteristic (ROC) curves and corresponding area under the curve (AUC) scores were used to compare discrimination effects between different influential factors for curve magnitude. A *P-*value < 0.05 was considered statistically significant. All data were analyzed using SPSS 25.0 (IBM Corp, Armonk, NY, USA) and the *R* programing language.

## Results

### Demographic characteristics of positive students stratified by curve magnitude

As shown in Table 1, the PPV was 55.4% for the maximal Cobb angle of 10–19°, 37.8% for 20–39°, and 2.6% for ≥ 40° in the 426 students who were screened as positive. Both our [20] and other studies [2] have previously depicted a higher incidence of AIS in women;, in the present analysis we further found that the proportion of women with AIS with different curve magnitudes was higher than that of males (Cobb angle 10–19°: 43.2 vs. 12.2%; 20–39°: 34.7 vs. 3.1%; ≥ 40°: 2.6 vs. 0.0%; *Z* = -3.929, *P* < 0.001), suggesting that women present with more severe scoliosis. Patients with AIS were mainly aged 7–12y (52.6%) and 13–15y (39.0%) with Cobb angles of 10–19° (31.9 and 21.8%) and 20–39° (19.5 and 16.0%), which are greater than those observed in adolescents aged 16–19y (*χ2* = 6.300, *P* = 0.043). These results indicate that the sex and age of patients with AIS are significantly associated with the severity of the curve magnitude.

**Table 1:** Demographics and incorrect postures of participants stratified by the distribution of the Cobb angle

Variables	Distribution of Cobb angle *n* (%)				Z^*^/χ^2#^	*P* Value
	< 10°	10–19°	20–39°	≥ 40°		
**Total**	18 (4.2)	236 (55.4)	161 (37.8)	11 (2.6)		
**Sex**					-3.929	**< 0.001**
Boys	4 (0.9)	52 (12.2)	13 (3.1)	0 (0.0)		
Girls	14 (3.3)	184 (43.2)	148 (34.7)	11 (2.6)		
**Age (years)**					6.300	**0.043**
7–12	14 (3.3)	136 (31.9)	83 (19.5)	5 (1.2)		
13–15	4 (0.9)	93 (21.8)	68 (16.0)	5 (1.2)		
16–19	0 (0.0)	7 (1.6)	10 (6.2)	1 (0.2)		
**Shoulder-height difference**					9.126	**0.010**
Normal	4 (0.9)	46 (10.8)	20 (4.7)	1 (0.2)		
Left shoulder height	9 (2.1)	100 (23.5)	60 (14.1)	4 (0.9)		
Right shoulder height	5 (4.2)	90 (21.1)	81 (19.0)	6 (1.4)		
**Scapula tilt**					8.048	**0.018**
Normal	5 (1.2)	48 (11.3)	22 (5.2)	2 (0.5)		
Tilt to the left	11 (2.6)	100 (23.5)	66 (15.5)	3 (0.7)		
Tilt to the right	2 (0.5)	88 (20.7)	73 (17.1)	6 (1.4)		
**Lumbar concave**					2.942	0.230
Normal	6 (1.4)	97 (59.9)	55 (34.0)	4 (0.9)		
Left concave	5 (1.2)	59 (13.8)	53 (12.4)	4 (0.9)		
Right concave	7 (1.6)	80 (18.8)	53 (12.4)	3 (0.7)		
**Pelvic tilt**					2.396	0.302
Normal	14 (3.3)	192 (45.1)	120 (28.2)	8 (1.9)		
Tilt to the left	3 (0.7)	15 (3.5)	16 (3.8)	1 (0.2)		
Tilt to the right	1 (0.2)	29 (6.8)	25 (5.9)	2 (0.5)		
**Flat back**					-1.427	0.154
Normal	18 (4.2)	232 (54.5)	161 (38.0)	11 (2.6)		
Abnormal	0 (0.0)	4 (0.9)	0 (0.0)	0 (0.0)		
**Rib hump**					12.721	**0.002**
Normal	4 (0.9)	97 (22.8)	47 (11.0)	1 (0.2)		
Tilt to the left	4 (0.9)	41 (9.6)	18 (4.2)	1 (0.2)		
Tilt to the right	10 (2.3)	98 (23.0)	96 (22.5)	9 (2.1)		
**Angle of thoracic rotation**					13.882	**0.001**
Normal (ATR: 0–4**°**)	6 (1.4)	129 (30.3)	59 (13.8)	1 (0.2)		
Left (ATR: ≥ 5**°**)	3 (0.7)	25 (5.9)	17 (4.0)	1(0.2)		
Right (ATR: ≥ 5**°**)	9 (2.1)	82 (19.2)	85 (20.0)	9 (2.1)		
**Thoracolumbar kyphosis**					4.400	0.111
Normal	18 (4.2)	224 (52.6)	152 (35.7)	9 (2.1)		
Tilt to the left	0 (0.0)	3 (0.7)	6 (1.4)	1 (0.2)		
Tilt to the right	0 (0.0)	9 (2.1)	3 (0.7)	1 (0.2)		
**Angle of thoracolumbar rotation**					2.324	0.313
Normal (ATR: 0–4**°**)	18 (4.2)	225 (52.8)	154 (36.2)	9 (2.1)		
Left (ATR: ≥ 5**°**)	0 (0.0)	3 (0.7)	4 (0.9)	1(0.2)		
Right (ATR: ≥ 5**°**)	0 (0.0)	8 (1.9)	3 (0.7)	1 (0.2)		
**Lumbar kyphosis**					2.711	0.258
Normal	4 (0.9)	69 (16.2)	55 (12.9)	4 (0.9)		
Tilt to the left	10 (2.3)	115 (27.0)	78 (18.3)	6 (1.4)		
Tilt to the right	4 (0.9)	52 (12.2)	28 (6.6)	1 (0.2)		
**Angle of lumbar rotation**					0.343	0.842
Normal (ATR: 0–4**°**)	9 (2.1)	97 (22.8)	64 (15.0)	5 (1.2)		
Left (ATR: ≥ 5**°**)	7 (1.6)	97 (22.8)	70 (16.4)	5 (1.2)		
Right (ATR: ≥ 5**°**)	18 (4.2)	42 (9.9)	27 (6.3)	1 (0.2)		

n, number; ATR, angle of trunk rotation

^*^ Mann-Whitney U test; ^#^Kruskal-Wallis test

The bold numbers of *P* value represent the significant differences

### Incorrect postures and ATR Associated with the curve magnitude

As shown in Table 1, incorrect postures, including shoulder height, scapula tilt, and rib hump, were significantly different among the different curve magnitude groups. The AIS patients with Cobb angles of 10–19°, 20–39°, and ≥ 40° had greater incidences of left shoulder height (23.5%, 14.1%, and 0.9%), right shoulder height (21.1%, 19.0%, and 1.4%), left scapula tilt (23.5%, 15.5%, and 0.7%), and right scapula tilt (20.7%, 17.1%, and 1.4%) than that of normal shoulder (10.8%, 4.7%, and 0.2%; *χ2* = 9.126, *P* = 0.010) and scapula height (11.3%, 5.2%, and 0.5%; *χ2* = 8.048, *P* = 0.018). Interestingly, patients with AIS with Cobb angles of 20–39° and ≥ 40° had a greater proportion of right rib hump (22.5% and 2.1%) than those with normal rib morphology (11.0% and 0.2%) and left rib hump (4.2% and 0.2%; *χ2* = 12.721, *P* = 0.002). Correspondingly, the AIS group with Cobb angles 20–39° and ≥ 40° had significantly higher frequencies of students with an angle of right thoracic rotation ≥ 5° (20.0 and 2.1%) compared to that of normal thoracic rotation 0–4° (13.8 and 0.2%) and left thoracic rotation ≥ 5° (4.0 and 0.2%; *χ2* = 13.882, *P* = 0.001). No significant differences were reported in the percentages of other incorrect postures (pelvic tilt, lumbar concave, flat back, thoracolumbar kyphosis, and lumbar kyphosis) or ATR (angle of thoracolumbar or lumbar rotation) in the different curve magnitude groups. Taken together, the incorrect postures, including shoulder-height difference, scapula tilt, a rib hump, and angle of thoracic rotation, were significantly associated with the curve magnitude in students with AIS.

### Multifactorial analysis of correlative factors of curve magnitude

Univariate LR analysis models were applied to explore the factors associated with the curve magnitude. The results showed that female gender (OR = 2.92, 95%CI 1.67–5.09, *P* < 0.001), age 16–19y (OR = 2.83, 95%CI 1.10–7.28, *P* = 0.031), right shoulder height (OR = 2.15, 95%CI 1.23–3.75, *P* = 0.007), right scapula tilt (OR = 2.03, 95%CI 1.18–3.50, *P* = 0.010), right rib hump (OR = 1.88, 95%CI 1.23–2.85, *P* = 0.003), right thoracic rotation ≥ 5° (OR = 2.14, 95%CI 1.43–3.20, *P* < 0.001), and left thoracolumbar kyphosis (OR = 3.79, 95%CI 1.06–13.56, *P* = 0.041) were all significantly associated with the severity of the curve magnitude (Table 2).

**Table 2:** Univariate logistic regression analysis of incorrect postures associated with spinal curve magnitude

Variables	OR	95%CI	*P* Value^*^
Sex			
Boys	1		
Girls	2.92	1.67–5.09	**< 0.001**
**Age (years)**			
7–12	1		
13–15	1.36	0.93–2.01	0.116
16–19	2.83	1.10–7.28	**0.031**
**Shoulder**-**height difference**			
Normal	1		
Left shoulder height	1.35	0.77–2.38	0.289
Right shoulder height	2.15	1.23–3.75	**0.007**
**Scapula tilt**			
Normal	1		
Tilt to the left	1.31	0.76–2.25	0.329
Tilt to the right	2.03	1.18–3.50	**0.010**
**Lumbar concave**			
Normal	1		
Left concave	1.47	0.93–2.34	0.100
Right concave	1.07	0.68–1.67	0.774
**Pelvic tilt**			
Normal	1		
Tilt to the left	1.33	0.67–2.62	0.413
Tilt to the right	1.48	0.86–2.56	0.161
**Flat back**			
Normal	1		
Abnormal	0.250	0.03–2.12	0.204
**Rib hump**			
Normal	1		
Tilt to the left	0.83	0.46–1.51	0.550
Tilt to the right	1.88	1.23–2.85	**0.003**
**Angle of thoracic rotation**			
Normal (ATR: 0–4**°**)	1		
Left (ATR: ≥ 5**°**)	1.28	0.67–2.42	0.455
Right (ATR: ≥ 5**°**)	2.14	1.43–3.20	**< 0.001**
**Thoracolumbar kyphosis**			
Normal	1		
Tilt to the left	3.79	1.06–13.56	**0.041**
Tilt to the right	0.87	0.29–2.60	0.795
**Angle of thoracolumbar rotation**			
Normal (ATR: 0–4**°**)	1		
Left (ATR: ≥ 5**°**)	3.04	0.75–12.28	0.199
Right (ATR: ≥ 5**°**)	0.95	0.31–2.96	0.933
**Lumbar kyphosis**			
Normal	1		
Tilt to the left	0.82	0.53–1.25	0.351
Tilt to the right	0.64	0.37–1.10	0.105
**Angle of lumbar rotation**			
Normal (ATR: 0–4**°**)	1		
Left (ATR: ≥ 5**°**)	1.12	0.75–1.69	0.576
Right (ATR: ≥ 5**°**)	1.01	0.59–1.74	0.960

OR, odds ratio; CI, confidence interval; ATR, angle of trunk rotation

^*^Ordered logistic regression analysis

The bold numbers of *P* value represent the significant differences

Furthermore, factors with a *P-*value < 0.05 in the univariate analysis were used for multivariate analysis. As shown in Table 3, women were more likely to develop a severe curve magnitude (AOR = 3.23, 95%CI 1.81–5.73, *P* < 0.001) than men; students aged 16–19y had 5.08 times (AOR = 5.08, 95%CI 1.86–13.91, *P* = 0.002) higher likelihood of a severe curve magnitude compared to those aged 7–12y. Interestingly, the presence of a right rib hump, but not left rib hump, was associated with a 1.72 times (AOR = 1.72, 95%CI 1.11–2.64, *P* = 0.015) higher likelihood of having severe curve magnitude than normal students. These results indicate that sex, age, and incorrect postures, especially a right rib hump, were significantly associated with a higher risk of developing severe AIS.

**Table 3:** Multivariate logistic regression analysis of incorrect postures associated with spinal curve magnitude

Variables	AOR	95%CI	*P* Value
Sex			
Boys	1		
Girls	3.23	1.81–5.73	**< 0.001**
**Age (years)**			
7–12	1		
13–15	1.47	0.99–2.19	0.059
16–19	5.08	1.86–13.91	**0.002**
**Shoulder-height difference**			
Normal	1		
Left shoulder height	0.97	0.41–2.30	0.944
Right shoulder height	1.22	0.52–2.85	0.651
**Scapula tilt**			
Normal	1		
Tilt to the left	1.26	0.55–2.92	0.585
Tilt to the right	1.54	0.67–3.53	0.313
**Rib hump**			
Normal	1		
Tilt to the left	0.76	0.41–1.40	0.380
Tilt to the right	1.72	1.11–2.64	**0.015**

AOR, adjusted odds ratio; CI, confidence interval

^*^Ordered logistic regression analysis

The bold numbers of *P* value represent the significant differences

### Compare the discrimination ability of influential factors for curve magnitude using ROC analysis

**Fig. 1:**
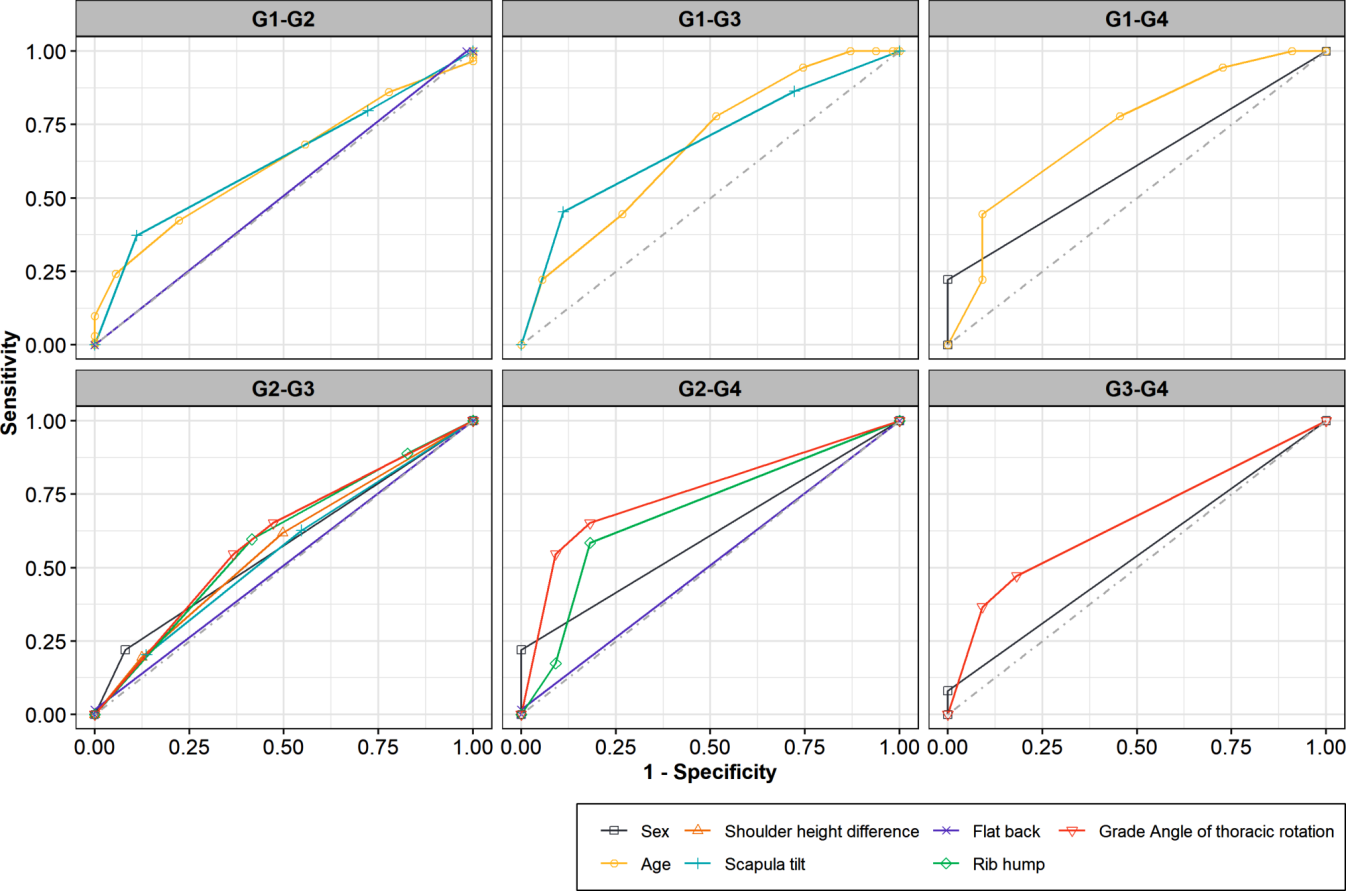
Results of ROC curve analysis by different risk factors for curve magnitude. ROC curve results for lumbar concave, pelvic tilt, thoracolumbar kyphosis, angle of thoracolumbar rotation, lumbar kyphosis, and angle of lumbar rotation were not shown because there was no statistical difference in their AUC value. G1: Cobb angle < 10°, G2: 10-19°, G3: 20-39°, G4: ≥ 40°

We employed ROC curves and AUC scores to compare the predictive effects of different influential factors for curve magnitude (Fig. 1). Similar to the results of LR models, sex, age, shoulder-height difference, scapula tilt, flat back, rib hump, angle of thoracic rotation could significantly distinguish different grades of major curve magnitude. The corresponding AUC scores were listed in Table S1.

## Discussion

Despite advancements in the SSS, current programs are still significantly limited in terms of a low PPV, excessive costs, and a high referral rate to radiography [12]. Our previous study discussed the effectiveness and feasibility of using incorrect postures as an indicator in SSS using a large scale population-based (595,057) dataset in China [20]. However, few prior studies have investigated the correlation between incorrect postures and the spinal curve magnitude. We analyzed the role of sex, age, incorrect postures, and ATR in identifying different curve magnitudes in 426 students with a full spine X-ray who were suspected of having scoliosis at our school screening. Our results showed that female gender, age 16–19y, and right rib hump may be the most reliable indicators of AIS severity.

The severity of AIS is known to correlate with sex and age. Although several studies from both our [19, 20] and other research groups [1, 2] have shown that sex and age are related to the incidence of AIS, few studies have verified their roles in the curve magnitude. We found that the proportion of women with AIS with different curve magnitudes (Cobb angle < 10°, 10–19°, 20–39°, ≥ 40°) was higher than that of men, indicating that AIS with different curve magnitudes occurred more frequently in women. In addition, our results showed that most students with AIS were aged 7–15y, and their Cobb angles were mainly 10–19° and 20–39°. More importantly, the LR analyses and ROC curves showed that female gender and age 16–19y were independently associated with the AIS severity. Although our results showed that students with AIS were mainly in the age groups of 7–12 or 13–15y, those aged 16–19y with suspected scoliosis may have more severe curve magnitudes. Furthermore, the AIS deformity progresses until skeletal maturity is reached [22]. Thus, scoliosis aggravates with increasing age in absence of early intervention, suggesting that once scoliosis is diagnosed, treatment should be conducted as soon as possible. The screening personnel should carefully explain the condition to the parents and recommend outpatient spine X-rays. These practices will improve the PPV and prevent further curve progression with early interventions.

The incorrect posture was associated with the AIS severity. Most of the studies on the occurrence of scoliosis were based on visual inspection of incorrect postures, such as scapular prominence, asymmetric shoulder height, and rib hump observed during the FBTs in SSS [23]. Some studies have shown that the trunk asymmetry does not significantly correlate with scoliosis [24, 25]. However, our previous studies have shown that the prevalence of incorrect posture was significantly higher in students diagnosed with AIS than in those with non-AIS [19, 20]. This study further explored the association between incorrect posture and curve magnitude. We found that the prevalence of shoulder-height difference, scapula tilt, and rib hump were significantly associated with a greater curve magnitude. Univariate LR analysis and ROC curves further showed that right shoulder height, right scapula tilt, right rib hump, and left thoracolumbar kyphosis were all related to the severity of spinal curvature. Moreover, multivariate LR analysis proved that right rib hump was independently associated with the severity of the curvature, showing that a right rib hump is an indication for screening of AIS severity in school programs. Several factors affect the efficacy of school screening programs [25]. Our team strongly recommends that incorrect postures be included in school screening programs to increase their effectiveness and minimize the negative effects of AIS on the students, families, and health system.

In our study, right thoracic rotation was associated with the severity of curve magnitude. ATR measurement using the scoliometer has been validated for scoliosis screening [26, 27], and our previous study [20] showed that thoracic, thoracolumbar, or lumbar rotation angles ≥ 5° indicated a higher risk for AIS than those with ATR < 5◦. However, few studies have explored the association between the curve magnitude and ATR. In this study, we found that the frequencies of right thoracic rotation ≥ 5° were significantly higher in the AIS group with Cobb angle 20–39° and ≥ 40° compared to that of thoracic rotation 0–4° and left thoracic rotation ≥ 5°. Moreover, univariate LR analysis and ROC curves revealed that right thoracic rotation ≥ 5° was a factor influencing severe curve magnitude. These findings supported the results of previous study [20], and further verified the correlation between right thoracic rotation ≥ 5° and severity of AIS, which could provide more accurate and effective assessment of students with scoliosis in a large-scale school screening.

This study has several limitations. First, it was difficult for the study to make causal inferences due to its cross-sectional design causing a potential for the reverse association. Second, due to the large volume of screening, other contributory factors (such as genetics, hormones, or nutritional status) associated with AIS were not adequately examined, perhaps exaggerating the extent of the link between improper posture and AIS severity. Third, the factors linked with the progression of AIS were not examined in this study.

## Conclusions

Despite these limitations, this is one of the first studies to demonstrate that sex, age, incorrect postures, and ATR are all associated with the curve magnitude of AIS among Chinese adolescents. This research also showed that right rib hump could be considered as the best indicator to evaluate the severity of scoliosis based on school and outpatient X-ray screening programs. Monitoring and early identification of incorrect postures and ATR could be a feasible and effective strategy to predict the severity of AIS and enable prompt intervention to prevent curve progression. Thus, students with incorrect posture and ATR, especially the right rib hump should rather be referred for a radiograph. Our team is currently conducting prospective cohort studies to further explore the long-term impact of incorrect posture on the progression of AIS.

### Electronic supplementary material

Below is the link to the electronic supplementary material.

Supplementary Material 1

## Data Availability

No datasets were generated or analysed during the current study.
