# Supplementary material for: Association between incorrect postures and curve magnitude of adolescent idiopathic scoliosis in china
Source: J Orthop Surg Res. 2024 May 17;19:300. doi: 10.1186/s13018-024-04767-z (PMC11100037; doi:10.1186/s13018-024-04767-z)
Supplement: Supplementary file 1 — Supplementary Material 1 [file 13018_2024_4767_MOESM2_ESM.doc]

**Table S1 AUC scores of different risk factors for curve magnitude**

| **Curve magnitude^∗^** | **Variables** | **AUC** | **95%CI** | ***P* Value** |  |
| --- | --- | --- | --- | --- | --- |
| G1-G4 | Sex | 0.61 | 0.51-0.71 | 0.028 |  |
| G2-G3 | Sex | 0.57 | 0.54-0.60 | < 0.001 |  |
| G2-G4 | Sex | 0.61 | 0.58-0.64 | < 0.001 |  |
| G3-G4 | Sex | 0.54 | 0.52-0.56 | < 0.001 |  |
| G1-G2 | Age | 0.62 | 0.51-0.74 | 0.034 |  |
| G1-G3 | Age | 0.68 | 0.55-0.80 | < 0.001 |  |
| G1-G4 | Age | 0.73 | 0.55-0.92 | 0.015 |  |
| G2-G3 | Shoulder height difference | 0.57 | 0.52-0.62 | < 0.001 |  |
| G1-G2 | Scapula tilt | 0.63 | 0.52-0.73 | 0.018 |  |
| G1-G3 | Scapula tilt | 0.69 | 0.58-0.79 | < 0.001 |  |
| G2-G3 | Scapula tilt | 0.55 | 0.50-0.61 | 0.049 |  |
| G1-G2 | Flat back | 0.51 | 0.50-0.52 | 0.044 |  |
| G2-G3 | Flat back | 0.51 | 0.50-0.52 | 0.044 |  |
| G2-G4 | Flat back | 0.51 | 0.50-0.52 | 0.044 |  |
| G2-G3 | Rib hump | 0.60 | 0.54-0.64 | < 0.001 |  |
| G2-G4 | Rib hump | 0.69 | 0.55-0.83 | 0.009 |  |
| G2-G3 | Angle of thoracic rotation | 0.60 | 0.55-0.65 | < 0.001 |  |
| G2-G4 | Angle of thoracic rotation | 0.76 | 0.65-0.86 | < 0.001 |  |
| G3-G4 | Angle of thoracic rotation | 0.66 | 0.54-0.77 | < 0.001 |  |

**^∗^**G1: Cobb angle < 10°, G2: 10–19°, G3: 20–39°, G4: ≥ 40°

AOR, adjusted odds ratio; CI, confidence interval.

^*^Ordered logistic regression analysis.

The bold numbers of *P* value represent the significant differences.
